# Cancer mortality by educational level in the city of Barcelona

**DOI:** 10.1038/sj.bjc.6690108

**Published:** 1999-02

**Authors:** E Fernandez, C Borrell

**Affiliations:** Institut Universitari de Salut Pública de Catalunya, Campus de Bellvitge, Universitat de Barcelona, L'Hospitalet (Barcelona), Catalonia, 08097, Spain; Unitat de Bioestadística, Departament de Salut Pública, Universitat de Barcelona, Barcelona, Catalonia, 08036, Spain; Institut Municipal de Salut Pública de Barcelona, Pça Lesseps 1, Barcelona, Catalonia, 08023, Spain

**Keywords:** socioeconomic status, cancer, mortality, education, inequalities, Spain

## Abstract

The objective of this study was to examine the relationship between educational level and mortality from cancer in the city of Barcelona. The data were derived from a record linkage between the Barcelona Mortality Registry and the Municipal Census. The relative risks (RR) of death and 95% confidence intervals (CIs) according to level of education were derived from Poisson regression models. For all malignancies, men in the lowest educational level had a RR of death of 1.21 (95% CI 1.13–1.29) compared with men with a university degree, whereas for women a significant decreasing in risk was observed (RR 0.81; 95% CI 0.74–0.90). Among men, significant negative trends of increasing risk according to level of education were present for cancer of the mouth and pharynx (RR 1.70 for lowest vs. highest level of education), oesophagus (RR 2.14), stomach (RR 1.99), larynx (RR 2.56) and lung (RR 1.35). Among women, cervical cancer was negatively related to education (RR 2.62), whereas a positive trend was present for cancers of the colon (RR 0.76), pancreas (RR 0.59), lung (RR 0.55) and breast (RR 0.65). The present study confirms for the first time, at an individual level, the existence of socioeconomic differences in mortality for several cancer sites in Barcelona, Spain. There is a need to implement health programmes and public health policies to reduce these inequities. © 1999 Cancer Research Campaign


*British Journal of Cancer* (1999) **79**(3/4), 684–689
© 1999 Cancer Research Campaign
Article no. bjoc.1998.0108

### Cancer mortality by educational level in the city of Barcelona

**E Fernandez[1,2] and C Borrell[3]**

[1]Institut Universitari de Salut Pública de Catalunya, Campus de Bellvitge, Universitat de Barcelona, Ctra. Feixa Llarga s/n, 08097 L'Hospitalet (Barcelona), Catalonia, Spain; [2]Unitat de Bioestadística, Departament de Salut Pública, Universitat de Barcelona, Carrer Casanovas 143, 08036 Barcelona, Catalonia, Spain; [3]Institut Municipal de Salut Pública de Barcelona, Pça Lesseps 1, 08023 Barcelona, Catalonia, Spain

**Summary** The objective of this study was to examine the relationship between educational level and mortality from cancer in the city of Barcelona. The data were derived from a record linkage between the Barcelona Mortality Registry and the Municipal Census. The relative risks (RR) of death and 95% confidence intervals (CIs) according to level of education were derived from Poisson regression models. For all malignancies, men in the lowest educational level had a RR of death of 1.21 (95% CI 1.13–1.29) compared with men with a university degree, whereas for women a significant decreasing in risk was observed (RR 0.81; 95% CI 0.74–0.90). Among men, significant negative trends of increasing risk according to level of education were present for cancer of the mouth and pharynx (RR 1.70 for lowest vs. highest level of education), oesophagus (RR 2.14), stomach (RR 1.99), larynx (RR 2.56) and lung (RR 1.35). Among women, cervical cancer was negatively related to education (RR 2.62), whereas a positive trend was present for cancers of the colon (RR 0.76), pancreas (RR 0.59), lung (RR 0.55) and breast (RR 0.65). The present study confirms for the first time, at an individual level, the existence of socioeconomic differences in mortality for several cancer sites in Barcelona, Spain. There is a need to implement health programmes and public health policies to reduce these inequities.

**Keywords:** socioeconomic status; cancer; mortality; education; inequalities; Spain

Social differences in cancer incidence and mortality have been extensively studied and documented in several countries. Overall, cancer mortality is inversely related to socioeconomic status among men, but not among women (Doll and Peto, 1981; Logan, 1982; OPCS, 1986; Colditz et al, 1996; Faggiano et al, 1997). Cancers of the mouth and pharynx, oesophagus, stomach, liver, larynx, lung and cervix are negatively related to socioeconomic status, whereas breast, large bowel, prostate, testis, ovary, melanoma, and leukaemia are positively associated with socioeconomic status (Pearce and Howard, 1986; Vagerö and Persson, 1987; Levi et al, 1988; Kogevinas, 1990; Davey et al, 1991; Williams et al, 1991; La Vecchia et al, 1992; Schrijvers and Mackenbach, 1994; Faggiano et al, 1995; Smith et al, 1996; Rosso et al, 1997).

Although two comprehensive reports (Regidor et al, 1994; Navarro and Benach, 1996) have provided evidence of social inequalities in health in Spain, few studies have addressed socioeconomic differences in mortality (Regidor and González, 1989; Casi and Moreno, 1992; Arias et al, 1993; Borrell and Arias, 1993, 1995). Only two of those studies, however, adopted an individual-based approach, rather than ecological designs (Regidor et al, 1995; Arias and Borrell, 1998). Individual-based studies are also scarce in the international literature, especially in southern Mediterranean countries (Faggiano et al, 1997). Incomplete data on occupation in Spanish death certificates, legal restrictions on record linkage at a national level between census information and death registries, and the absence of a National Death Index for epidemiological studies (Benavides et al, 1989; Gispert, 1989; García Benavides et al, 1991) have been suggested as reasons for the lack of individual-based studies in investigations of socioeconomic inequalities in mortality in Spain (Álvarez-Dardet et al, 1995; Regidor et al, 1995).

Based on death certificates of men from eight Spanish provinces, Regidor et al (1995) reported an excess mortality of cancers of the lung and of the stomach among 'production and transportation workers' compared with 'professional and managerial' occupations, whereas no difference was apparent for large bowel mortality (Regidor et al, 1996*a*). Arias and Borrell (1998) found that illiterate men were at higher risk of dying from lung cancer, whereas no association was present for large bowel cancer. For women, positive non-significant trends in risk were observed between educational level and lung and breast cancer. However, to date, no comprehensive study has systematically analysed mortality in several cancer sites.

Thus, to provide further evidence on this issue, we have analysed the relationship between educational level, used as an indicator of socioeconomic status, and mortality from cancer (all causes and specific sites) in the city of Barcelona by means of a record linkage analysis between the Municipal Death Registry and the Census.

*Received 19 May 1998*
*Revised 3 July 1998*
*Accepted 14 July 1998*

*Correspondence to:* E Fernandez, Institut Universitari de Salut Pública de Catalunya, Campus de Bellvitge, Universitat de Barcelona, Ctra. Feixa Llarga s/n, 08097 L'Hospitalet (Barcelona), Catalonia, Spain

### MATERIAL AND METHODS

The study base consisted of the 18 153 deaths from malignancies among the 64 721 deaths in subjects aged ≥25 years old occurring

**Table 1** Number of deaths and person–years at risk (population ≥25 years) according to the level of education: men, Barcelona, Spain, 1992–95

| Cancer site (ICD-9) | Level of education | | | | |
|---|---|---|---|---|---|
| | University | High school | Middle school | Primary school | Less than primary school |
| All sites (140–208) | 1180 | 971 | 1210 | 3381 | 3466 |
| Mouth and pharynx (140–149) | 39 | 31 | 41 | 125 | 126 |
| Oesophagus (150) | 21 | 24 | 28 | 126 | 85 |
| Stomach (151) | 46 | 43 | 70 | 209 | 221 |
| Colon (153) | 118 | 73 | 104 | 260 | 303 |
| Rectum (154) | 30 | 22 | 26 | 85 | 95 |
| Liver (155) | 67 | 72 | 82 | 233 | 226 |
| Pancreas (157) | 52 | 38 | 58 | 99 | 105 |
| Larynx (161) | 22 | 28 | 37 | 104 | 111 |
| Lung (162) | 319 | 275 | 344 | 963 | 1006 |
| Skin (172–173) | 26 | 14 | 12 | 28 | 26 |
| Prostate (185) | 118 | 78 | 104 | 288 | 322 |
| Urinary bladder (188) | 76 | 45 | 66 | 196 | 246 |
| Nervous system (191–192) | 23 | 23 | 29 | 52 | 45 |
| Lymphoma (200–203) | 48 | 46 | 46 | 113 | 108 |
| Leukaemia (204–208) | 33 | 27 | 28 | 96 | 72 |
| Person–years | 388 992 | 344 632 | 345 024 | 580 296 | 365 492 |

**Table 2** Number of deaths and person–years at risk (population ≥25 years) according to the level of education: women, Barcelona, Spain, 1992–95

| Cancer site (ICD-9) | Level of education | | | | |
|---|---|---|---|---|---|
| | University | High school | Middle school | Primary school | Less than primary school |
| All sites (140–208) | 463 | 344 | 629 | 2325 | 3036 |
| Stomach (151) | 23 | 12 | 30 | 131 | 238 |
| Colon (153) | 49 | 35 | 72 | 287 | 357 |
| Rectum (54) | 9 | 10 | 21 | 69 | 109 |
| Liver (155) | 21 | 10 | 23 | 146 | 187 |
| Pancreas (157) | 25 | 14 | 31 | 102 | 142 |
| Lung (162) | 34 | 30 | 37 | 119 | 141 |
| Skin (172–173) | 3 | 4 | 7 | 25 | 25 |
| Breast (174) | 136 | 83 | 155 | 490 | 525 |
| Cervix uteri (180) | 6 | 8 | 14 | 38 | 63 |
| Corpus uteri (179, 182) | 9 | 19 | 21 | 86 | 106 |
| Ovary (183) | 24 | 17 | 35 | 98 | 134 |
| Urinary bladder (188) | 3 | 10 | 9 | 48 | 73 |
| Nervous system (191–192) | 13 | 7 | 17 | 57 | 73 |
| Lymphoma (200–203) | 22 | 10 | 31 | 128 | 179 |
| Leukaemia (204–208) | 11 | 14 | 24 | 75 | 108 |
| Person–years | 337 552 | 254 024 | 375 924 | 789 440 | 667 944 |

in the city of Barcelona during the years 1992–95. The data were derived from the record linkage between the Barcelona Mortality Registry and the 1991 Barcelona Municipal Census. Cause of death was obtained from death certificates, coded using the International Classification of Diseases 9th revision (ICD-9) (WHO, 1977). The 18 153 subjects with ICD-9 codes corresponding to malignancies (ICD-9 codes 140–208) were selected (28% of total mortality). The cancer sites selected (according to statistical power considerations) are listed in Table 1 for men and Table 2 for women, with the corresponding ICD-9 codes. Deaths among Barcelona residents that occurred outside the city were gathered from the Mortality Registry maintained by the Ministry of Health of Catalonia. All confidentiality rules concerning management of individual data from official registries were complied with. Level of education was assigned according to the highest educational level attained as declared at the 1991 Municipal Census, and was categorized in five categories as follows: university degree (>15 years of school education), high school (11–14 years), middle school (8 years), primary studies or elementary school (5 years), and less than primary school education (less than 5 years).

Relative risks (RRs) of death and 95% confidence intervals (CIs) for all cancer sites together and for selected cancer sites were derived from Poisson regression models (Breslow and Day, 1987), in which the logarithm of the mortality rate was the dependent variable and educational level the explanatory variable with

*© Cancer Research Campaign 1999*

**Table 3** Relative risk (RRs) of death and 95% confidence interval (CI) according to level of education: men, Barcelona, 1992–95

| | Level of education | | | | | | |
|---|---|---|---|---|---|---|---|
| **Cancer site (ICD-9)** | **University[a]** | **High school** | **Middle school** | **Primary school** | **Less than primary school** | **$\chi^2$ for trend** | ***P*-value** |
| All sites (140–208) | 1 | 1.18 (1.09–1.29) | 1.20 (1.11–1.30) | 1.18 (1.10–1.26) | 1.21 (1.13–1.29) | 22.0 | 0.001 |
| Mouth and pharynx (140–149) | 1 | 1.14 (0.71–1.83) | 1.19 (0.77–1.85) | 1.44 (1.00–2.06) | 1.70 (1.18–2.45) | 10.6 | 0.001 |
| Oesophagus (150) | 1 | 1.64 (0.91–2.94) | 1.53 (0.87–2.69) | 2.74 (1.72–4.35) | 2.14 (1.32–3.48) | 14.3 | 0.001 |
| Stomach (151) | 1 | 1.35 (0.89–2.05) | 1.79 (1.24–2.60) | 1.88 (1.36–2.58) | 1.99 (1.44–2.74) | 20.1 | 0.001 |
| Colon (153) | 1 | 0.88 (0.66–1.18) | 1.02 (0.78–1.33) | 0.88 (0.71–1.10) | 1.05 (0.85–1.30) | 0.2 | 0.621 |
| Rectum (154) | 1 | 1.07 (0.62–1.85) | 1.03 (0.61–1.74) | 1.17 (0.77–1.77) | 1.29 (0.85–1.96) | 1.9 | 0.163 |
| Liver (155) | 1 | 1.52 (1.09–2.13) | 1.41 (1.02–1.95) | 1.37 (1.04–1.80) | 1.33 (1.01–1.75) | 1.4 | 0.224 |
| Pancreas (157) | 1 | 1.05 (0.69–1.60) | 1.30 (0.90–1.90) | 0.78 (0.55–1.09) | 0.84 (0.60–1.17) | 3.6 | 0.055 |
| Larynx (161) | 1 | 1.79 (1.02–3.13) | 1.92 (1.13–3.25) | 2.11 (1.33–3.35) | 2.56 (1.62–4.06) | 16.6 | 0.001 |
| Lung (162) | 1 | 1.23 (1.05–1.45) | 1.24 (1.07–1.45) | 1.24 (1.09–1.41) | 1.35 (1.19–1.53) | 17.6 | 0.001 |
| Skin (172–173) | 1 | 0.75 (0.39–1.47) | 0.55 (0.28–1.10) | 0.53 (0.31–0.90) | 0.53 (0.30–0.93) | 6.1 | 0.013 |
| Prostate (189) | 1 | 0.99 (0.75–1.32) | 1.11 (0.85–1.44) | 0.94 (0.76–1.17) | 0.90 (0.73–1.12) | 1.6 | 0.192 |
| Urinary bladder (188) | 1 | 0.84 (0.58–1.22) | 1.04 (0.75–1.44) | 1.02 (0.78–1.33) | 1.20 (0.92–1.55) | 3.6 | 0.055 |
| Nervous system (191–192) | 1 | 1.33 (0.74–2.38) | 1.43 (0.83–2.48) | 1.07 (0.65–1.76) | 1.09 (0.65–1.82) | 0.03 | 0.853 |
| Lymphoma (200–203) | 1 | 1.30 (0.87–1.95) | 1.12 (0.75–1.68) | 1.02 (0.72–1.43) | 0.99 (0.70–1.40) | 0.4 | 0.495 |
| Leukaemia (204–208) | 1 | 1.17 (0.70–1.95) | 1.01 (0.61–1.68) | 1.21 (0.81–1.80) | 0.89 (0.59–1.35) | 0.2 | 0.587 |

[a]Reference category.

**Table 4** Relative risk (RRs) of death and 95% confidence interval (CI) according to level of education: Women, Barcelona, 1992–95

| | Level of education | | | | | | |
|---|---|---|---|---|---|---|---|
| **Cancer site (ICD-9)** | **University[a]** | **High school** | **Middle school** | **Primary school** | **Less than primary school** | **$\chi^2$ for trend** | ***P*-value** |
| All sites (140–208) | 1 | 0.99 (0.86–1.14) | 0.99 (0.88–1.12) | 0.86 (0.78–0.95) | 0.82 (0.74–0.90) | 30.3 | 0.001 |
| Stomach (151) | 1 | 0.70 (0.35–1.40) | 0.96 (0.56–1.65) | 0.88 (0.56–1.38) | 1.07 (0.69–1.66) | 1.60 | 0.205 |
| Colon (153) | 1 | 0.96 (0.62–1.48) | 1.07 (0.75–1.55) | 0.91 (0.67–1.23) | 0.76 (0.56–1.03) | 7.2 | 0.007 |
| Rectum (154) | 1 | 1.48 (0.60–3.64) | 1.70 (0.78–3.71) | 1.18 (0.59–2.37) | 1.27 (0.64–2.52) | 0.00 | 0.992 |
| Liver (155) | 1 | 0.60 (0.28–1.28) | 0.78 (0.43–1.40) | 1.01 (0.64–1.60) | 0.90 (0.57–1.42) | 0.06 | 0.802 |
| Pancreas (157) | 1 | 0.75 (0.39–1.45) | 0.90 (0.53–1.52) | 0.63 (0.40–0.98) | 0.59 (0.38–0.91) | 7.1 | 0.008 |
| Lung (162) | 1 | 1.17 (0.72–1.91) | 0.78 (0.49–1.24) | 0.61 (0.41–0.89) | 0.55 (0.37–0.81) | 17.3 | 0.001 |
| Skin (172–173) | 1 | 0.70 (0.28–1.77) | 0.95 (0.46–1.96) | 0.88 (0.47–1.62) | 0.93 (0.51–1.73) | 0.01 | 0.914 |
| Breast (174) | 1 | 0.82 (0.62–1.08) | 0.83 (0.66–1.05) | 0.73 (0.60–0.89) | 0.65 (0.53–0.79) | 20.4 | 0.001 |
| Cervix uteri (180) | 1 | 1.81 (0.63–5.21) | 1.84 (0.70–4.79) | 1.68 (0.70–4.05) | 2.62 (1.09–6.27) | 5.2 | 0.022 |
| Corpus uteri (179, 182) | 1 | 2.82 (1.28–6.24) | 1.70 (0.78–3.70) | 1.54 (0.77–3.08) | 1.34 (0.67–2.67) | 1.0 | 0.309 |
| Ovary (183) | 1 | 0.93 (0.50–1.74) | 1.05 (0.62–1.77) | 0.76 (0.48–1.20) | 0.83 (0.53–1.30) | 1.1 | 0.276 |
| Urinary bladder (188) | – | 1[b] | 0.91 (0.39–2.12) | 0.89 (0.48–1.65) | 0.82 (0.45–1.50) | 0.4 | 0.487 |
| Nervous system (191–192) | 1 | 0.70 (0.28–1.77) | 0.95 (0.46–1.96) | 0.88 (0.47–1.62) | 0.93 (0.51–1.73) | 0.01 | 0.914 |
| Lymphoma (200–203) | 1 | 0.60 (0.28–1.27) | 1.03 (0.59–1.77) | 0.93 (0.59–1.77) | 0.92 (0.58–1.44) | 0.01 | 0.935 |
| Leukaemia (204–208) | 1 | 1.76 (0.79–3.87) | 1.67 (0.81–3.40) | 1.19 (0.63–2.26) | 1.18 (0.62–2.22) | 0.3 | 0.549 |

[a]Referent category; [b]Referent category is University + High School.

university degree as the reference category. Because previous studies had indicated that the association between socioeconomic status and cancer mortality is different according to gender, all models were fitted separately for men and women. All models included age at death in decades to allow for potential confounding. Stratified analyses according to age (three categories: 25–44, 45–64, ≥65 years old) were conducted, and interaction terms for age and level of education (five categories) were also assessed. All models showed acceptable fit ascertained by the likelihood ratio statistic or deviance (Breslow and Day, 1987). Linear trends on risk were based on the $\chi^2$ test for trend, computed as the difference between the deviance of the model with and without level of education in an ordinal coding form (Breslow and Day, 1987).

### RESULTS

Record linkage between the Mortality Registry and the Census was successful in 93.7% of cases and, thus, educational level could be assigned to 10 208 men and 6797 women, who were finally included for analysis. Absolute numbers for all cancer deaths and each of the 18 cancers or cancer groups considered, together with the person–years at risk for each educational level are shown in Table 1 for men and Table 2 for women. The commonest causes of cancer death among men was lung (2907 cases, 28.5% of deaths) followed by colorectal (1116 cases, 10.9%) cancers; and among women, breast (1389 cases, 20.4%) and colorectal (1018 cases, 15.0%) cancers.

For all malignancies, men in the lowest educational level had a RR of death of 1.21 (95% CI 1.13–1.29) compared with men with

 *© Cancer Research Campaign 1999*

**Table 5** Relative risk (RRs) of death and 95% confidence interval (CI) according to level of education, by age groups and sex, Barcelona, 1992–95

| Sex | Age group (years) | Level of education: University[a] | High school | Middle school | Primary school | Less than primary school | *P*-value for interaction |
|---|---|---|---|---|---|---|---|
| Men | | | | | | | |
| | 25–44 | 1 | 0.93 (0.65–1.32) | 1.25 (0.89–1.76) | 1.61 (1.17–2.23) | 2.97 (1.93–4.57) | |
| | 45–64 | 1 | 1.35 (1.17–1.57) | 1.25 (1.09–1.45) | 1.42 (1.26–1.60) | 1.63 (1.45–1.85) | |
| | ≥65 | 1 | 1.09 (0.98–1.22) | 1.14 (1.03–1.27) | 1.08 (0.99–1.17) | 1.15 (1.06–1.25) | <0.001 |
| Women | | | | | | | |
| | 25–44 | 1 | 0.93 (0.64–1.37) | 0.94 (0.66–1.35) | 1.12 (0.80–1.57) | 1.04 (0.59–1.82) | |
| | 45–64 | 1 | 0.82 (0.65–1.05) | 0.83 (0.68–1.02) | 0.77 (0.65–0.92) | 0.80 (0.68–0.96) | |
| | ≥65 | 1 | 1.03 (0.85–1.25) | 1.08 (0.92–1.28) | 0.96 (0.84–1.10) | 0.98 (0.86–1.13) | 0.489 |

[a]Referent category.

a university degree, with a significant trend of increasing mortality with decreasing level of education (Table 3). Among women, cancer mortality showed a significant direct trend. Women with less than primary studies were at less risk of cancer death than women with a university degree (RR 0.81; 95% CI 0.74–0.90) (Table 4).

Among men, significant negative trends of increasing risk according to level of education were present for cancer of the mouth and pharynx (RR 1.70 lowest vs. highest level of education), cancer of the oesophagus (RR 2.14), cancer of the stomach (RR 1.99), cancer of the larynx (RR 2.56) and cancer of the lung (RR 1.35), whereas cancer of the skin showed a significant positive trend (RR 0.53) (Table 3). Among women (Table 4), cancer of the cervix uteri was negatively related to education (RR 2.62), whereas the trend in risk for cancer of the colon (RR 0.76), pancreas (RR 0.59), lung (RR 0.55) and breast (RR 0.66) significantly decreased with lower level of education.

The association between education and cancer mortality was further analysed in separate strata of age (25–44, 45–64, ≥65 years). Among men, for all cancer sites, a similar negative pattern was present in the three age groups, but was more pronounced in the 25–44 age group (*P* for interaction <0.001, Table 5). Among women, the positive association was only apparent for those women aged 45–64 years (Table 5). For specific cancer sites, no variation in the pattern of risk was apparent according to age, except for lung cancer in men, with a stronger association between low level of education and mortality in the youngest age group (25–44 years old) (RR 3.91; 95% CI 1.83–8.35).

### DISCUSSION

The opportunity to link two data sources, the Municipal Census and the Municipal Mortality Registry, has permitted the study of socioeconomic differences in mortality across several cancer sites for the first time at an individual level in Spain. The results of the present investigation are consistent with most of the US and European literature and confirm that less educated men are at higher risk of death from cancers of the mouth and pharynx, oesophagus, stomach and lung. The same is true for cancer of the cervix uteri among women. As in certain other studies, mortality from skin cancer (mainly melanoma) was reduced in men with the lowest level of education, likewise pancreas, lung and breast cancer mortality in women with the lowest level of education (Doll and Peto, 1981; Logan, 1982; Pearce and Howard, 1986; Vagerö and Persson, 1987; Levi et al, 1988; Davey et al, 1991; Williams et al, 1991; Faggiano et al, 1995, 1997).

Environmental and lifestyle factors that influence cancer occurrence are associated with socioeconomic status. Thus, differences in the prevalence of known or likely risk factors for cancer such as alcohol consumption, tobacco smoking, dietary habits or physical activity across the educational groups may explain part but not all of the differences in mortality observed (Marmot et al, 1984; Feldman et al, 1989; Davey Smith et al, 1994). Socioeconomic differences, however, persist even after adjustment for the known risk factors, and neither the processes determining risk exposure nor the mechanisms by which exposures produce disease are well understood (Marmot et al, 1984; Feldman et al, 1989; Davey Smith et al, 1994; Dorlie et al, 1995; Marmot and Feeney, 1997).

Mouth, pharynx and oesophagus cancer are directly related to excessive alcohol consumption (IARC, 1988), which is more prevalent among less educated men in our country (Borrell et al, 1995; Gutiérrez-Fisac, 1995; Navarro and Benach, 1996). The pattern observed for lung cancer mortality among men (negative) and women (positive) reflects smoking habits in this population, in which smoking is more prevalent in lower social classes in men but until recently in higher social classes in women (Borrell et al, 1995; Navarro and Benach, 1996; Nebot et al, 1996).

Breast cancer mortality was positively associated with education and may be related to delayed childbearing or other differential patterns in reproductive and hormonal factors such as parity or age at menarche and menopause (Kelsey and Horn-Ross, 1993). Early detection of breast cancer might account for some of the differences observed because in the city of Barcelona (Borrell et al, 1995; Rohlfs et al, 1998), as well as elsewhere in Spain (Luengo et al, 1996), more educated women or those in the highest socioeconomic groups are more likely to undergo mammography and, hence, to have reduced mortality. Such an effect in mortality cannot, however, be detected from these data because the population-based programme for breast cancer screening in the city of Barcelona was implemented in 1994 and does not cover the whole city, but only two districts (30% of susceptible women) (Pla de Salut, 1994; Rohlfs et al, 1998).

Recent observations in the US and in Europe indicate a reduction in the differentials in breast cancer mortality according to socioeconomic status (Lund and Jacobsen, 1991; Wagener and Schatzkin, 1994; Heck et al, 1997), attributed in part to a reduction or variation in risk factors for breast cancer across socioeconomic groups (Heck et al, 1997).

*© Cancer Research Campaign 1999*

Cancer of the cervix shows a negative pattern of association with education that may be attributable to a higher exposure to known risk factors including papillomavirus infection among less educated women (de Sanjosé et al, 1996), and also to differences in screening procedures according to socioeconomic status (Borrell et al, 1995; Bailie and Bourne, 1996; Sánchez et al, 1997). In Spain, there are no organized cervical cancer screening programmes, and cervical cytology is performed on a case-finding basis (Sánchez et al, 1997).

Cancer of the colon and rectum has been both negatively and positively linked in different studies to higher socioeconomic status groups both in men and women (Faggiano et al, 1997). In the present dataset, a negative association was found for colon cancer in women but not in men, and no association was found for rectal cancer.

Some limitations and strengths of this investigation deserve attention. Education has been used as a surrogate of socio-economic status, instead of social class based on occupation as commonly used in Europe. Social class classification has some limitations, such as the classification of non-working individuals (including unemployed and retired) and women, whereas the level of education is readily applied to these groups and does not change during adult life (Liberatos et al, 1988; Álvarez-Dardet et al, 1995; Berkman and Macintyre, 1997). Bias due to lack of information on educational level is unlikely, given the almost complete linkage between the Census and the Mortality Registry (93.7%). Deaths with no information for level of education occurred principally in individuals under 45 years of age (Arias, 1996). In our population, however, the probability of dying from cancer below 45 years of age is extremely low, with a cumulative risk of 0.83% for men and 0.68% for women (Moreno et al, 1997).

Because level of education was gathered from the census both for deceased individuals and for the population at risk, this study is, moreover, free from the 'numerator/denominator bias' that operates when mortality rates according to socioeconomic status are computed from different sources in the numerator (from death certificates) and in the denominator (from the census) (Islley and Baker, 1991). Finally, the acceptable validity of the underlying cause of death from death certificates in the city of Barcelona is also reassuring against information bias (Pañella et al, 1989; Montellà et al, 1995).

In conclusion, the present study confirms the existence of socioeconomic differences in mortality for several cancers in Barcelona, an issue on which little research has been performed in Spain. These observations have implications for both public health programmes and research. With respect to public health programmes, there is a need in Spain to implement policies which promote equity in health (Regidor et al, 1994; Navarro and Benach, 1996; Navarro, 1997; Regidor, 1997). Although social inequalities in the use of National Health Service facilities (primary and hospital care) have almost disappeared in Spain, health differentials (e.g. in self-perceived health) by socioeconomic status are still present (Navarro and Benach, 1996; Navarro, 1997; Regidor et al, 1996*b*). In terms of research, monitoring and evaluation of public health policies aimed at reducing inequalities is essential. Further studies on the causes of social differences in cancer mortality are also required to understand these inequities and to implement public health policies. The data presented here also suggest that reducing inequalities in education would have important public health implications. In the same way that a decline in mortality from most infectious diseases was due to general improvements in hygiene, income and nutrition rather than treatment or prevention of specific biological agents, it is likely that social and economic changes that determine lifestyles and exposure to risk factors will lead to advances in cancer prevention (Pearce, 1996).

### ACKNOWLEDGEMENTS

The authors are grateful to Enrique Regidor, Miquel Porta, Vicenç Navarro, Andreu Segura, Michael Herdman and Luis Rajmil for their critiques of earlier versions of the paper. Preliminary results of this investigation were presented at the XV Meeting of the Spanish Society of Epidemiology, Oviedo, 24–26 September 1997 [*Gac Sanit* (1997) **11** (suppl. 1): 39].

### REFERENCES

Álvarez Dardet C, Alonso J, Domingo A and Regidor E (1995) *La Medición de la Clase Social en Ciencias de la Salud*. SG Editores-Sociedad Española de Epidemiologia: Barcelona

Arias LC (1996) Desigualdades en la mortalidad según la educación en la ciudad de Barcelona (Master of Public Health thesis). Institut Universitari de Salut Pública de Catalunya: Barcelona

Arias LC and Borrell C (1998) Desigualdades en la mortalidad según la educación en la ciudad de Barcelona. *Med Clin (Barc)* **110**: 161–166

Arias A, Rebagliato M, Palumbo MA, Bellver R, Ashton J, Colomer C, Costa J, Flynn P and Alvarez-Dardet C (1993) Desigualdades en salud en Barcelona y Valencia. *Med Clin (Barc)* **100**: 281–287

Bailie RS and Bourne D (1996) Surveillance for equity in cervical cytology screening. *Int J Epidemiol* **25**: 46–51

Benavides FG, Bolumar F and Peris R (1989) Quality of death certificates in Valencia, Spain. *Am J Public Health* **79**: 1352–1354

Berkman LF and Macintyre S (1997) The measurement of social class in health studies: old measures and new formulations. In *Social Inequalities and Cancer*. Kogevinas M, Pearce N, Susser M and Boffeta P (eds.), pp. 51–64. IARC Scientific Publications no. 138: Lyon

Borrell C and Arias A (1993) Desigualtats en mortalitat en els barris de Barcelona, 1983–89. *Gac Sanit* **7**: 205–220

Borrell C and Arias A (1995) Socioeconomic factors and mortality in urban settings: the case of Barcelona, Spain. *J Epidemiol Community Health* **49**: 460–465

Borrell C, Pasarín I and Plasència A (1995) *Enquesta de Salut de Barcelona 1992–93*. Estadístiques de Salut No. 23. Ajuntament de Barcelona: Barcelona

Breslow NE and Day NE (1987) The analysis of cohort studies. In *Statistical Methods in Cancer Research, Vol. II*, pp. 119–176. IARC Scientific Publications no. 82: Lyon

Casi A and Moreno C (1992) Desigualdades ante la muerte: estudio comparativo entre comunidades de Navarra en el segmento de población de 25 a 74 años. *Aten Primaria* **10**: 543–548

Colditz G, Dejong W, Hunter D, Trichopoulos D and Willet W (1996) *Harvard Report on Cancer Prevention*, Volume 1, Socioeconomic status. *Causes of Human Cancer*. *Cancer Causes Control* **7**: S33–S35

Davey SG, Leon D, Shipley MJ and Rose G (1991) Socio-economic differentials in cancer among men. *Int J Epidemiol* **20**: 339–345

Davey Smith G, Blane D and Bartley M (1994) Explanations for socio-economic differentials in mortality. Evidence from Britain and elsewhere. *Eur J Public Health* **4**: 131–144

de Sanjosé S, Bosch FX, Muñoz N, Tafur L, Gili M, Izarzugaza I, Izquierdo A, Navarro C, Vergara A, Muñoz MT, Ascunce N and Shah KV (1996) Socioeconomic differences in cervical cancer: two case–control studies in Colombia and Spain. *Am J Public Health* **86**: 1532–1538

Doll R and Peto R (1981) The causes of cancer. Quantitative estimates of avoidable risks of cancer in the United States today. *J Natl Cancer Inst* **66**: 1191–1308.

Dorlie PD, Backlund E and Keller JB (1995) US mortality by economic, demographic, and social characteristics: the National Longitudinal Study. *Am J Public Health* **85**: 949–956

Faggiano F, Lemma P, Costa G, Gnavi R and Pagnarelli F (1995) Cancer mortality by educational level in Italy. *Cancer Causes Control* **6**: 311–320

Faggiano F, Partanen T, Kogevinas M and Boffeta P (1997) Socioeconomic differences in cancer incidence and mortality. In *Social Inequalities and*

 © Cancer Research Campaign 1999

*Cancer*. Kogevinas M, Pearce N, Susser M and Boffeta P (eds.), pp. 65–206. IARC Scientific Publications No. 138: Lyon

Feldman JJ, Makuc DM, Kleinman JC and Cornoni-Huntley J (1989) National trends in educational differentials in mortality. *Am J Epidemiol* **129**: 919–933

García Benavides F, Segura Benedicto A and Godoy Laserna C (1991) Estadísticas de mortalidad en España: pequeños problemas, grandes perspectivas. *Revisiones Salud Publica* **2**: 43–66

Gispert R (1989) La variable professió en les estadístiques de mortalitat. *Gac Sanit* **3**: 371–376

Gutiérrez-Fisac JL (1995) Indicadores de consumo de alcohol en España. *Med Clin (Barc)* **104**: 544–550

Heck KE, Wagener DK, Schatzkin A, Devesa SS and Breen N (1997) Socioeconomic status and breast cancer mortality, 1989 through 1993: an analysis of education data from death certificates. *Am J Public Health* **87**: 1218–1222

IARC (1988) Alcohol drinking. In *IARC Monographs on the Evaluation of the Carcinogenic Risk of Chemicals to Humans, Vol. 44.* IARC: Lyon

Islley R and Baker D (1991) Contextual variations in the meaning of health inequality. *Soc Sci Med* **32**: 103–109

Kelsey JL and Horn-Ross PL (1993) Breast cancer: magnitude of the problem and descriptive epidemiology. *Epidemiol Rev* **15**: 7–16

Kogevinas E (1990) *Longitudinal Study: Socio-demographic Differences in Cancer Survival, 1971–1983.* OPCS series LS No. 5. Her Majesty's Stationery Office: London

La Vecchia C, Negri E and Franceschi S (1992) Education and cancer risk. *Cancer* **70**: 2935–2941

Levi F, Negri E, La Vecchia C and TE VC (1988) Socioeconomic groups and cancer risk at death in the Swiss canton of Vaud. *Int J Epidemiol* **17**: 711–717

Liberatos P, Link BG and Kelsey JL (1988) The measurement of social class in epidemiology. *Epidemiol Rev* **10**: 87–121

Logan WPD (1982). *Cancer Mortality by Occupation and Social Class.* Studies on medical and population subjects No. 44. Her Majesty's Stationery Office: London

Luengo S, Lázaro P, Madero R, Alviro F, Fitch K, Azcona B, Perez JM and Caballero P (1996) Equity in the access to mammography in Spain. *Soc Sci Med* **43**: 1263–1271

Lund E and Jacobsen BK (1991) Education and breast cancer mortality: experience from a large Norwegian cohort study. *Cancer Causes Control* **2**: 235–238

Marmot M and Feeney A (1997) General explanations for social inequalities in health. In *Social Inequalities and Cancer*. pp. 207–228. Kogevinas M, Pearce N, Susser M and Boffeta P (eds.). IARC Scientific Publications no. 138: Lyon

Marmot MG, Shipley MJ and Rose G (1984) Inequalities in death – specific explanations of a general pattern? *Lancet* **1**: 1003–1006

Montellà N, Ricart I, Borrell C, Clos R and Caylà JA (1995) Comparación de las defunciones del registro de casos de SIDA y de las defunctiones del registro de mortalidad. Barcelona 1991–1992. *Rev San Hig Publ* **69**: 49–58

Moreno V, Sánchez V, Galceran J, Borràs JM, Borràs J and Bosch FX (1997) Riesgo de enfermar y morir por cáncer en Cataluña. *Med Clin (Barc)* **110**: 86–93

Navarro V (1997) The 'Black Report' of Spain – The Commission on Social Inequalities in Health. *Am J Public Health* **87**: 334–335

Navarro V and Benach J (eds) (1996) *Desigualdades Sociales en Salud en España*. Ministerio de Sanidad y Consumo: Madrid

Nebot M, Borrell C, Ballestín M and Villalbí JR (1996) Prevalencia y características asociadas al consumo de tabaco en población general en Barcelona entre 1983 y 1992. *Rev Clin Esp* **196**: 359–364

Office of Population Censuses and Surveys (1986) *Occupational Mortality 1979–80, 1982–83, Decennial Supplement.* Her Majesty's Stationery Office: London

Pañella H, Borrell C, Rodríguez C and Roca J (1989) Validación de la causa básica de defunción en Barcelona, 1985. *Med Clin (Barc)* **92**: 129–134

Pearce N (1996) Why study socioeconomic factors and cancer? In *Social Inequalities and Cancer*. Kogevinas M, Pearce N, Susser M and Boffeta P (eds.), pp. 17–24. IARC Scientific Publications no. 138: Lyon

Pearce NE and Howard JK (1986) Occupation, social class and male cancer mortality in New Zealand, 1974–1978. *Int J Epidemiol* **15**: 456–462

Pla de Salut de la regió sanitària Barcelona Ciutat 1993–1995 (1994) Ajuntament de Barcelona, Servei Català de la Salut: Barcelona

Regidor E (1997) Investigación y acción sobre las desigualdades en salud. *Med Clin (Barc)* **108**: 784–790

Regidor E and González J (1989) Desigualdad social y mortalidad en España. *Rev San Hig Publ* **63**: 107–116

Regidor E, Gutiérrez-Fisac JL and Rodríguez C (1994) *Differencias y Desigualdades en Salud en España*. Díaz de Santos: Madrid

Regidor E, Gutiérrez-Fisac JL and Rodríguez C (1995) Increased socioeconomic differences in mortality in eight Spanish provinces. *Soc Sci Med* **6**: 801–807

Regidor E, Gutiérrez-Fisac JL and Rodríguez C (1996*a*) Diferencias socioeconómicas en mortalidad en ocho provincias españolas. *Med Clin (Barc)* **106**: 285–289

Regidor E, de Mateo S, Gutiérrez-Fisac JL, Fernández de la Hoz K and Rodríguez C (1996*b*) Diferencias socioeconómicas en la utilización y accesibilidad de los servicios sanitarios en España. *Med Clin (Barc)* **107**: 285–288

Rohlfs I, Borrell C, Plasència A and Pasarín I (1998) Social inequalities and realisation of opportunistic screening mammographies in Barcelona (Spain). *J Epidemiol Community Health* **52**: 205–206

Rosso S, Faggiano F, Zanetti R and Costa G (1997) Social class and cancer survival in Turin, Italy. *J Epidemiol Community Health* **51**: 30–34

Sánchez V, Rohlfs I, Borràs JM and Borrell C (1977) Migration within Spain, level of education, and cervical cancer screening. *Eur J Cancer Prev* **6**: 31–37

Schrijvers CTM and Mackenbach JP (1994) Cancer patient survival by socioeconomic status in seven countries: a review for six common cancer sites. *J Epidemiol Community Health* **48**: 441–446

Smith D, Taylor R and Coates M (1996) Socioeconomic differentials in cancer incidence and mortality in urban New South Wales, 1987–1991. *Aust NZ J Public Health* **20**: 129–137

Vagerö D and Persson G (1987) Cancer survival and social class in Sweden. *J Epidemiol Community Health* **41**: 204–209

Wagener DK and Schatzkin A (1994) Temporal trends in the socioeconomic gradient for breast cancer mortality among US women. *Am J Public Health* **84**: 1003–1006

Williams J, Clifford C, Hopper J and Giles G (1991) Socio-economic status and cancer incidence in Melbourne. *Eur J Cancer* **27**: 917–921

World Health Organization (1977) *Manual of International Classification of Diseases, Injuries and Causes of Death*, 9th Revision. WHO: Geneva

© Cancer Research Campaign 1999
